# Semi-supervised machine learning for automated species identification by collagen peptide mass fingerprinting

**DOI:** 10.1186/s12859-018-2221-3

**Published:** 2018-06-26

**Authors:** Muxin Gu, Michael Buckley

**Affiliations:** 1 Michael Smith Building, Faculty of Biology, Medicine and Health, The University of Manchester, Manchester, M13 9PT UK; 2 Manchester Institute of Biotechnology, School of Earth and Environmental Sciences, The University of Manchester, 131 Princess Street, Manchester, M1 7DN UK

**Keywords:** Collagen fingerprinting, Ancient bone identification, High-throughput species identification, Species biomarker identification, PCA, Hierarchical clustering

## Abstract

**Background:**

Biomolecular methods for species identification are increasingly being utilised in the study of changing environments, both at the microscopic and macroscopic levels. High-throughput peptide mass fingerprinting has been largely applied to bacterial identification, but increasingly used to identify archaeological and palaeontological skeletal material to yield information on past environments and human-animal interaction. However, as applications move away from predominantly domesticate and the more abundant wild fauna to a much wider range of less common taxa that do not yet have genetically-derived sequence information, robust methods of species identification and biomarker selection need to be determined.

**Results:**

Here we developed a supervised machine learning algorithm for classifying the species of ancient remains based on collagen fingerprinting. The aim was to minimise requirements on prior knowledge of known species while yielding satisfactory sensitivity and specificity. The algorithm uses iterations of a modified random forest classifier with a similarity scoring system to expand its identified samples. We tested it on a set of 6805 spectra and found that a high level of accuracy can be achieved with a training set of five identified specimens per taxon.

**Conclusions:**

This method consistently achieves higher accuracy than two-dimensional principal component analysis and similar accuracy with hierarchical clustering using optimised parameters, which greatly reduces requirements for human input. Within the vertebrata, we demonstrate that this method was able to achieve the taxonomic resolution of family or sub-family level whereas the genus- or species-level identification may require manual interpretation or further experiments. In addition, it also identifies additional species biomarkers than those previously published.

**Electronic supplementary material:**

The online version of this article (10.1186/s12859-018-2221-3) contains supplementary material, which is available to authorized users.

## Background

### Biomolecular species identification

Knowing the species from which a sample derives can be highly informative of the environment, whether this is at the microscopic or macroscopic scale. In the case of microorganisms this can be important to understand processes of infection [1–3] and/or decay [4, 5], whereas in the case of animals it can be important for understanding the effects of climate change or human impacts on biodiversity [6–8], or targeted at wildlife crime [9, 10]. For reasons relating to either difficulties in identification or practicalities of analysing high numbers of samples, molecular methods are often preferred over morphological approaches, the most common being those that utilise DNA [11]. Although DNA-based methods will undoubtedly continue to improve [12], there are alternative methods that utilise proteins, coded by DNA but still informative of species. These protein-based methods, such as those that generate peptide mass fingerprints (PMFs) via proteomic techniques, often do not have as much taxonomic resolution as DNA-based approaches, but can be subjected to much greater levels of high-throughput processing, capable of analysing thousands of samples in as little as a week. Another advantage is that proteins, particularly bone collagen, are known to survive for greater lengths of time than DNA [13], the identifications from which could be useful for inferring animal-human interactions or palaeobiodiversity change deeper into the past.

Fast production of PMFs by high-throughput soft-ionization mass spectrometry, particularly Matrix Assisted Laser Desorption Ionization (MALDI) Time of Flight (ToF) mass spectrometry, calls for automated decision-making systems for species assignment. The simplest strategy is to use biomarkers, which are peptides within the PMF that are characteristic of a taxonomic group. In microbial identification, biomarker-based methods were able to reach the species level with high accuracy in both bacteria and yeasts [14]. However, their performance in ancient species identification was less satisfactory due to difficulties in finding well-defined biomarkers not affected by great variations due to differences in levels of decay over time, greatly reducing relative concentration; ancient collagen, the main target of PMFs derived from archaeological and palaeontological specimens can contain many post-translational modifications (PTMs), some of which are also affected by decay. Therefore, previous studies have tended to combine biomarker-based methods with manual correction in order to improve performance [15, 16]. In recent studies, focus has shifted towards using information on the entire spectrum rather than specific markers. For example, Hollemeyer et al. [17] introduced the calculation of Euclidean distances between samples to separate distantly related groups and then used biomarkers to fine-tune the species assignment. In addition, multivariate regressions such as principal component analysis and partial least square regression have been used in addition to biomarkers to separate different taxa [15, 16].

### Machine learning

The above examples are part of the methodology known as expert systems, which implement the strategies and logic used by an experienced researcher for making decisions (e.g. using the presence/absence of manually identified biomarkers or applying certain cut-offs to hierarchical clustering trees). However, building expert systems can be difficult. For example, finding the logic orders to construct the decision trees requires extensive work examining a comprehensive set of PMFs, which often requires additional sequencing information to lend confidence to the homology of the markers. Moreover, the output of expert systems tends to be binary rather than probabilistic. In recent years, progress has been made towards more robust systems that can learn to become experts through a training process analogous to human learning - this approach is also known as machine learning.

Supervised machine learning uses a training set of samples with predetermined classes. For example, the training set of MALDI data can be represented as an *n* × *m* matrix *T* ∈ *ℝ*^*n* × *m*^, where *n* is the number of sample vectors in the training set and *m* is number of features in each vector (e.g. the presence/absence of biomarkers) and a class vector$\overset{\rightharpoonup}{\boldsymbol{c}}$that indicates the desired classification result. The classification algorithm learns to build a classifier that puts all training samples into the right class, or formally a function *f* such that $f\left(\overset{\rightharpoonup}{T_i}\right)={c}_i$ for *i* ∈ {1, 2…*n*}. Then the classifier *f* is applied to the real dataset *X* ∈ *ℝ*^*p* × *m*^ with *p* samples. One potential problem here is overfitting, which means that the classifier *f* only works for the training set but not the real set and this is why a separate validation set is often used to filter out bad classifiers. Another potential problem is the use of a single classifier. For example, on a small training set of four spectra with two of each species, many biomarkers could be able to distinguish the two species by chance and will not work on the real set. In fact, it is recognised that using a collection of classifiers generally has enhanced performance compared to any of its constituent classifiers [18–20]. This is also known as ensemble learning, which looks for *k* possible classifiers *f*_1_…*f*_*k*_ that satisfy the training set and constructs a meta-classifier ${M}_{f_1\dots {f}_k}$ to achieve boosted performance.

Widely used ensemble approaches include boosting and bagging. Boosting refers to the step-wise strategy that fixes incorrect classifications every time a new classifier is incorporated [21]. The other approach, bagging, also known as Bootstrap Aggregating, features random sampling from the original dataset and is more robust against overfitting than boosting [22, 23]. The main representative of bagging approaches is random forest, where *k* subsets of *s* dimensions *S*_1_…*S*_k_ ∈ *ℝ*^*n* × *s*^ are randomly drawn from the *m*-dimensional training set and decision trees *f*_1_…*f*_*k*_ are calculated for each subset. The final classifier is constructed by a majority vote from all decision trees [22]. More recently, various modifications on the original random forest algorithm have been made to enhance the performance or customise individual studies [24, 25].

The aim of this study was to use machine learning to build an automated algorithm for species identification on large MALDI datasets with minimal requirement of human input. We used data from a set of recent publications on the species identification of bone fragments from Pin Hole Cave by collagen fingerprinting, an important archaeological site in the UK that contains collections spanning approximately 40,000 years of intermittent human occupation. The main obstacles were that 1) noises in MALDI spectra due to chemical decay, 2) limited number of samples that can be used as the training set, and 3) the training set may not always span all species in the entire data. Here we tested a modified random forest algorithm on a large set of 6805 MALDI spectra. Starting with a small set of manually verified spectra from within the larger dataset, the algorithm progressively learns to improve its classification strategy and eventually becomes able to classify the entire dataset with high discovery rates and few errors.

## Methods

### Acquisition of MALDI-ToF mass spectrometry data

Mass spectrometry data were acquired from a previous publication [26], where microfaunal specimens were recovered from a single archaeological site called Pin Hole Cave (UK), with additional specimens from the spoil heap and elsewhere in the cave. A total set of 13,022 specimens were previously interpreted manually for species biomarkers of particular taxa (predominantly megafauna). Experimental protocols were exactly the same as previously published [26].

### Pre-processing of MALDI data

**Fig. 1:**
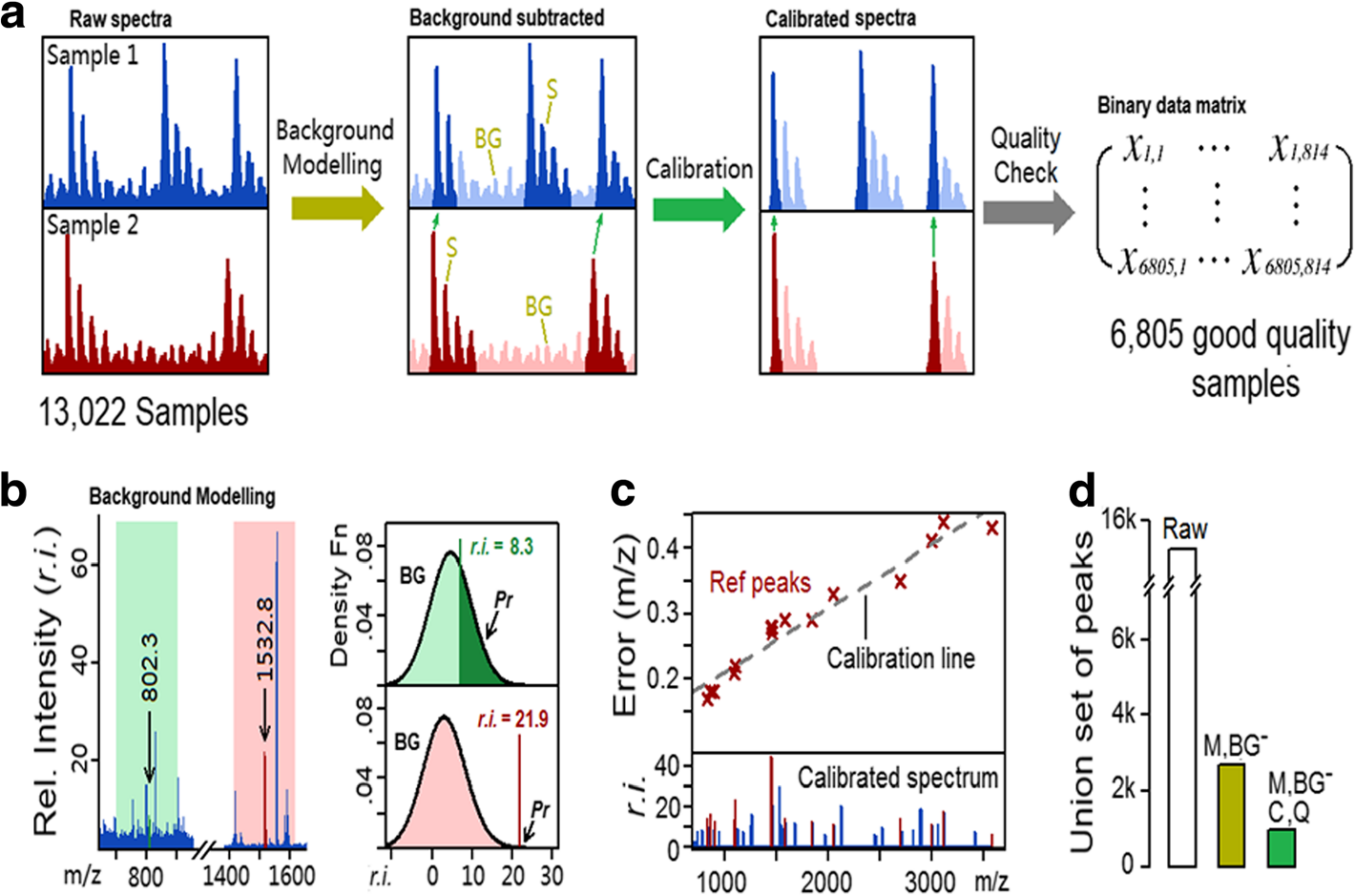
Flow chart of data pre-processing pipeline: (**a**) *m/z* peaks from MALDIquant were background-modelled, calibrated, quality checked and combined into a binary data matrix, (**b**) distribution of top 50% peaks within − 100 to + 100 *m/z* of the target peaks were modelled by a normal distribution; peaks with background probability (*Pr*) > 1 × 10^− 15^ were discarded (green and red areas give examples of background and signal respectively), (**c**) monoisotopic *m/z* values were matched to a reference set and linear models fitted between the errors and *m/z*; all peaks in the spectrum were subsequently corrected according to linear model, and (**d**) an illustration of the extent to which the ‘union’ set of peaks was greatly reduced by monoisotopic selection (M) and background subtraction (BG^−^) and further reduced by calibration (C) and quality check (Q)

With an initial set of 13,022 spectra (PMFs) from MALDI experiments [26, 27], the first step was to convert each PMF into a binary vector representing the presence or absence of *m/z* peaks (summarised in Fig. 1a). The R package MALDIquant was used to identify peak lists of *m/z* ratios and intensities for samples. Since MALDIquant has a permissive threshold, an extra step of filtering was applied to remove background noises. To determine whether a peak is noise, local background was modelled by extracting the intensities of all peaks within − 100 to + 100 *m/z* from the peak, removing the top 50% that were potential signals and fitting a normal function to the remaining peaks. Based on the normal function, likelihood of this peak for being noise was evaluated; peaks with likelihood > 1× 10^− 15^ were discarded and the signal peaks were extracted from the spectra (Fig. 1b).

Despite on-plate calibration, peaks from many samples remained off-calibrated by up to ±0.5 *m/z* units. Therefore, additional calibration was performed by comparing samples with a set of 50 most abundant peaks as reference (Additional file 1: Table S1). Calibration was omitted for samples where all peaks are within ±0.1 *m/z* units to reference. For each sample where the maximum error to reference was > 0.1 *m/z* units, a linear model was fitted between *m/z* values and errors within its spectrum:

$$ {Err}_{(M)}=k\cdotp M+b $$

where *Err* is the error of *m/z* between the spectrum and reference, *M* is the *m/z* ratio and *k* and *b* are coefficients for the linear model. The errors were then subtracted from *m/z* values for each peak to obtain a set of calibrated *m/z* values (Fig. 1c). From each cluster of peaks, the monoisotopic peak was extracted. Peaks that are within 2–3 *m/z* units were distinguished from isotopic effects by examining their relative intensities (Additional file 2: Figure S1). Spectra with poor quality (manually selected as < 6 peaks above 2000 *m/z* units) were excluded, leaving 6805 considered of good quality for this purpose. The pre-processing greatly reduced the redundancy and inaccuracy in the total set of peaks present in the datasets; the set of over 15,000 peaks across all raw spectra was reduced to ~ 5000 (including ~ 3000 monoisotopic) by background filtering and was further reduced to 814 monoisotopic peaks after calibration (Fig. 1d; Additional file 3: Table S2). These distinct peak bins were then combined into a 6805 × 814 matrix *X*, where *x*_*i*, *j*_ ∈ {0, 1} for any *i* and *j*:

$$ X=\left[\begin{array}{ccc}{x}_{1,1}& \cdots & {x}_{\mathrm{1,814}}\\ {}\vdots & \ddots & \vdots \\ {}{x}_{6805,1}& \cdots & {x}_{6805,814}\end{array}\right] $$

### Statistical analysis

Sensitivity and specificity of machine-learning classifiers were calculated as:

$$ Sensitivity=\frac{TP}{TP+ FN}\kern3.25em Specificity=\frac{TN}{TN+ FP} $$

where TP and TN stand for true positive and true negative and FP and FN stand for false positive and false negative respectively. Values of TP, TN, FP and FN were obtained by examining the overlaps between the positives/negatives identified by the classifier with the positives/negatives of the expanded validation set, which consists of the original validation set [26] and newly identified samples in this study that are manually checked for species. The sensitivity and specificity of hierarchical clustering and PCA were calculated using the same method.

## Results

### Model design for semi-supervised learning

**Fig. 2:**
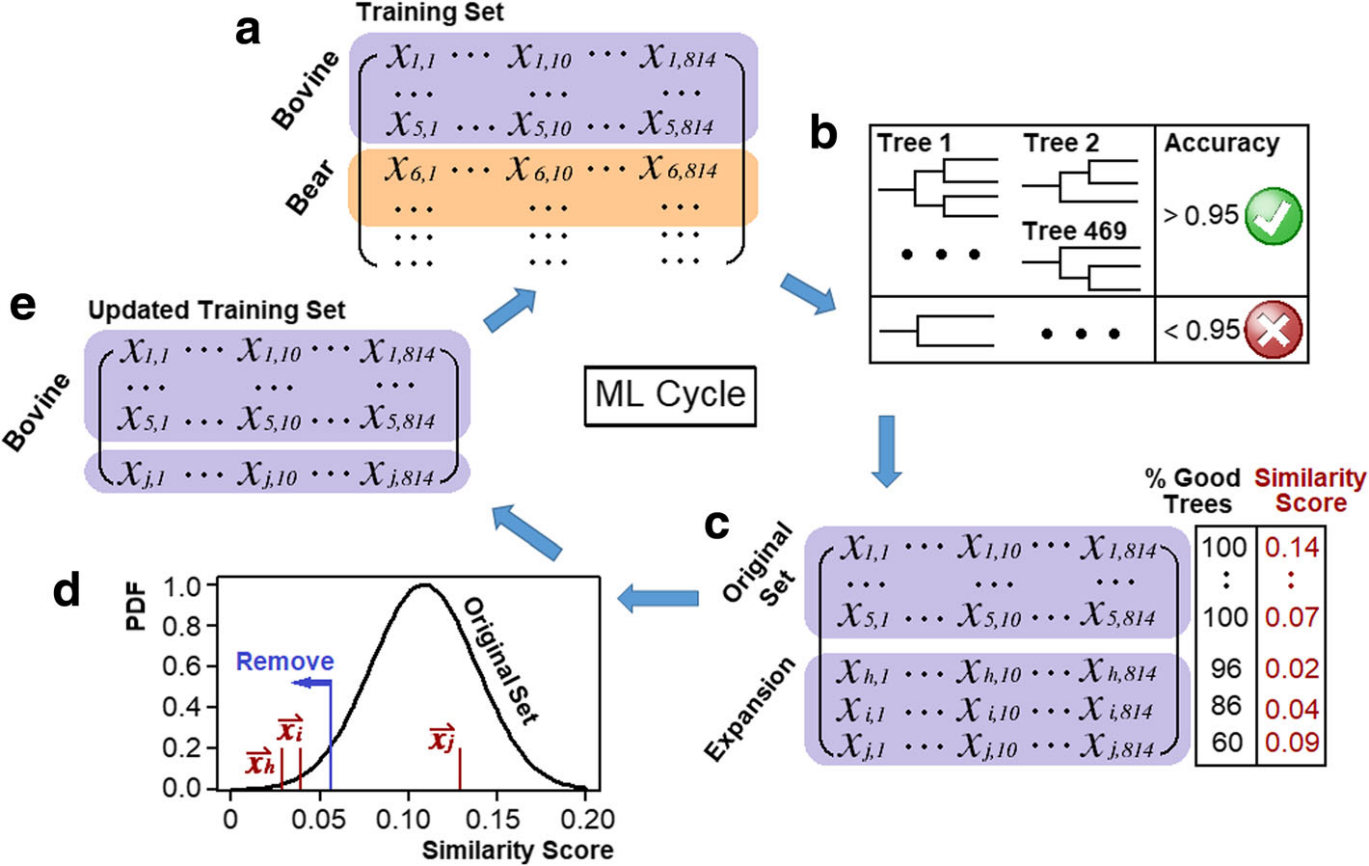
Cycle of semi-supervised learning model: (**a**) starting with a training set, each species within the training set undergoes B-E, where (**b**) is the use of random forest to draw 2000 subsets with 10 *m/z* peaks for the training set, from which the ID3 algorithm was used to find the optimal decision tree to separate the taxon from the rest (retaining those with accuracy > 0.95), (**c**) reflects majority voting of samples satisfying > 60% of trees, which were then added to the taxon, (**d**) the removal of samples significantly different to the training set (newly added samples with likelihood < 0.2 were removed) and (**e**) the updated set of samples were passed on to the next cycle as the new training set

As the aim was to identify species for the entire dataset with prior knowledge of only a small subset of samples, an iterative approach based on the random forest algorithm was developed. Each cycle starts with a training set of *n* samples (e.g. five for Cycle 1) for each taxon (Fig. 2a), for which 2000 subsets consisting of 10 peaks were randomly selected out of the 814 peaks. On each subset, the ID3 algorithm was applied to compute the optimal decision tree (Fig. 2b). All the decision trees with an accuracy > 95% were selected for majority voting and samples that passed > 60% of the votes were added to the expanded set of this species (Fig. 2c). In the rare case where a sample was voted positive by more than one taxon, the sample will be regarded as unclassified.

However, passing this expanded set to the next cycle could be problematic. The training set was unlikely to cover all species in the Pin Hole dataset and thus the expanded set could potentially contain undesired species. To tackle this problem, a filtering step was implemented to remove samples that are substantially different from the taxon. First, the characteristic vector $\overset{\rightharpoonup }{{\boldsymbol{v}}_{\boldsymbol{T}}}$ for the taxon was calculated as the difference of the fraction of a peak in this taxon and half of the maximum fraction of this peak in any other taxa:

$$ {\left(\overset{\rightharpoonup }{v_T}\right)}_p=\frac{1}{n}\sum \limits_{i\in T}{x}_{i,p}-0.5\times \underset{T^{\prime}\ne T}{\max}\left(\ \frac{1}{n}\sum \limits_{i\in {T}^{\prime }}{x}_{i,p}\ \right) $$

where *p* is the peak of the *p*^*th*^ element of $\overset{\rightharpoonup }{{\boldsymbol{v}}_{\boldsymbol{T}}}$, *x* is the binary value in matrix, *T* is the taxon and *T’* represent all the other taxa. The characteristic vector reflects both the uniqueness of peaks to this taxon and the pattern of all peaks in this taxon. A similarity score was then calculated as the inner product between a sample $\overset{\rightharpoonup }{{\boldsymbol{x}}_{\boldsymbol{i}}}$ and the taxon’s$\overset{\rightharpoonup }{{\boldsymbol{v}}_{\boldsymbol{T}}}$. To remove newly added samples that are vastly different to this taxon, a normal distribution was fitted to similarity scores of the original set and samples with a probability density < 0.2 were removed (Fig. 2d). The above process was repeated for all taxa in the training set and the new training set was passed on to the next iteration (Fig. 2e).

### Machine learning predicts species with high accuracy

**Fig. 3:**
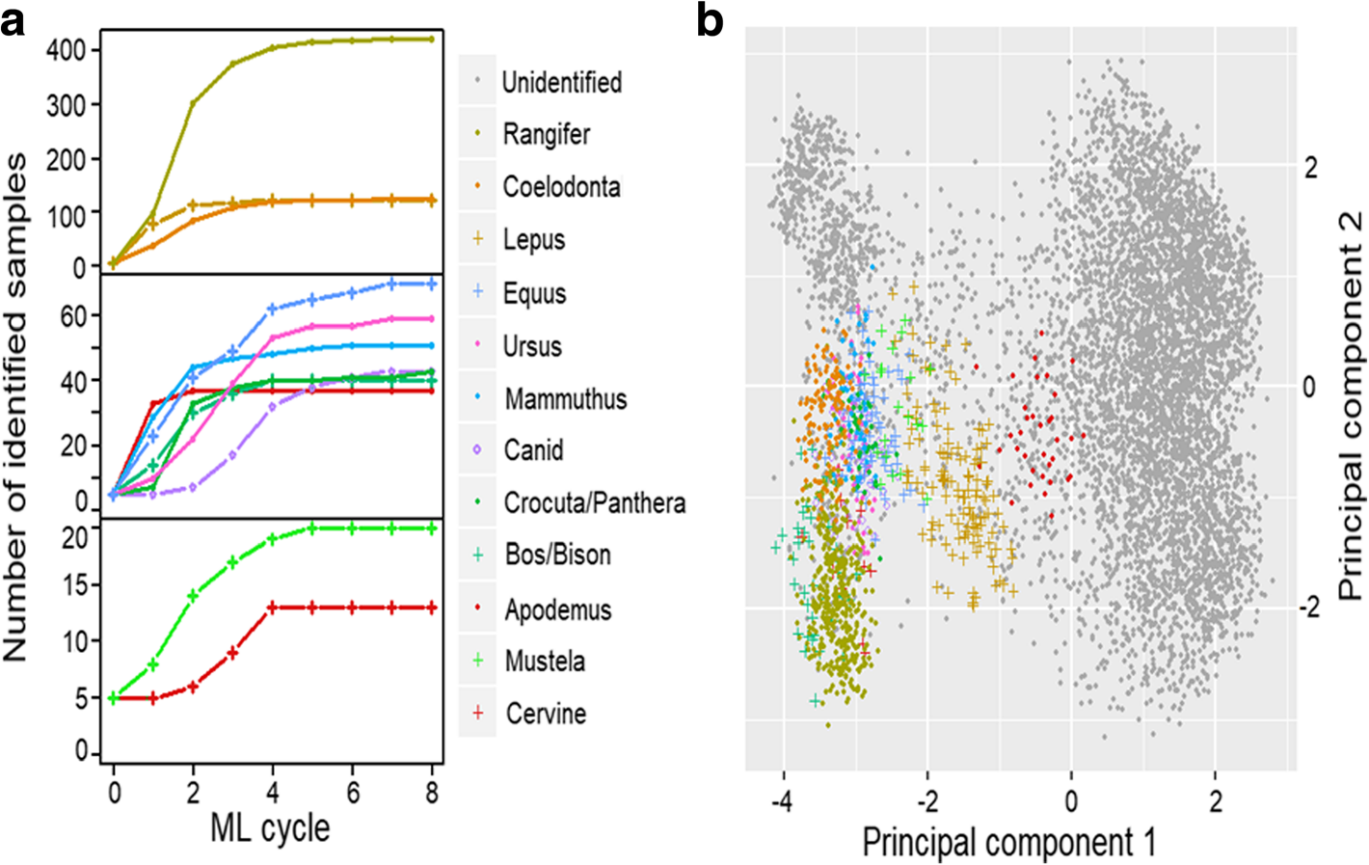
Output of machine learning cycles showing (**a**) the numbers of identified species increasing with each cycle of machine learning, generally converging within 8 cycles, and (**b**) visualisation of ML output where taxa are highlighted on the scatter plot between first and second principal components

We started machine learning (ML) using the validation set of 14 megafaunal taxa identified in Buckley et al. [26], including 37 bear (*Ursus*), 34 bovine (*Bos/Bison*), 48 horse (*Equus*), 15 hyaena (*Crocuta*), seven lion (*Panthera*), 76 hare (*Lepus*), 28 mammoth (*Mammuthus*), 13 red fox (*Vulpes*), eight arctic fox (*Alopex*), eight wolf (*Canis*), 13 weasel (*Mustela*), 308 reindeer (*Rangifer*), six roe deer (*Cervine*) and 82 rhinoceros (*Coelodonta*) samples, along with 11 field mouse (*Apodemus*) samples. Five samples were randomly drawn from each taxon and used as the training set for Cycle 1. Since ML struggled to distinguish between phylogenetically closely related species (Additional file 2: Figure S2), we pooled hyaenas with lions (denoted as *Crocuta/Panthera*) and red foxes, arctic foxes with wolves (denoted as *Canid*). Through iterations of ML, we observed increasing numbers of identified samples in each taxon and the numbers converged to constants within eight cycles (Fig. 3a, Additional file 4: Table S3). Each taxon tended to occupy a distinct domain on the multivariate plot of the first two principal components (Fig. 3b). We observed no clear boundaries between taxa, which is as expected since the principal component alone is insufficient in separating different taxa.

**Fig. 4:**
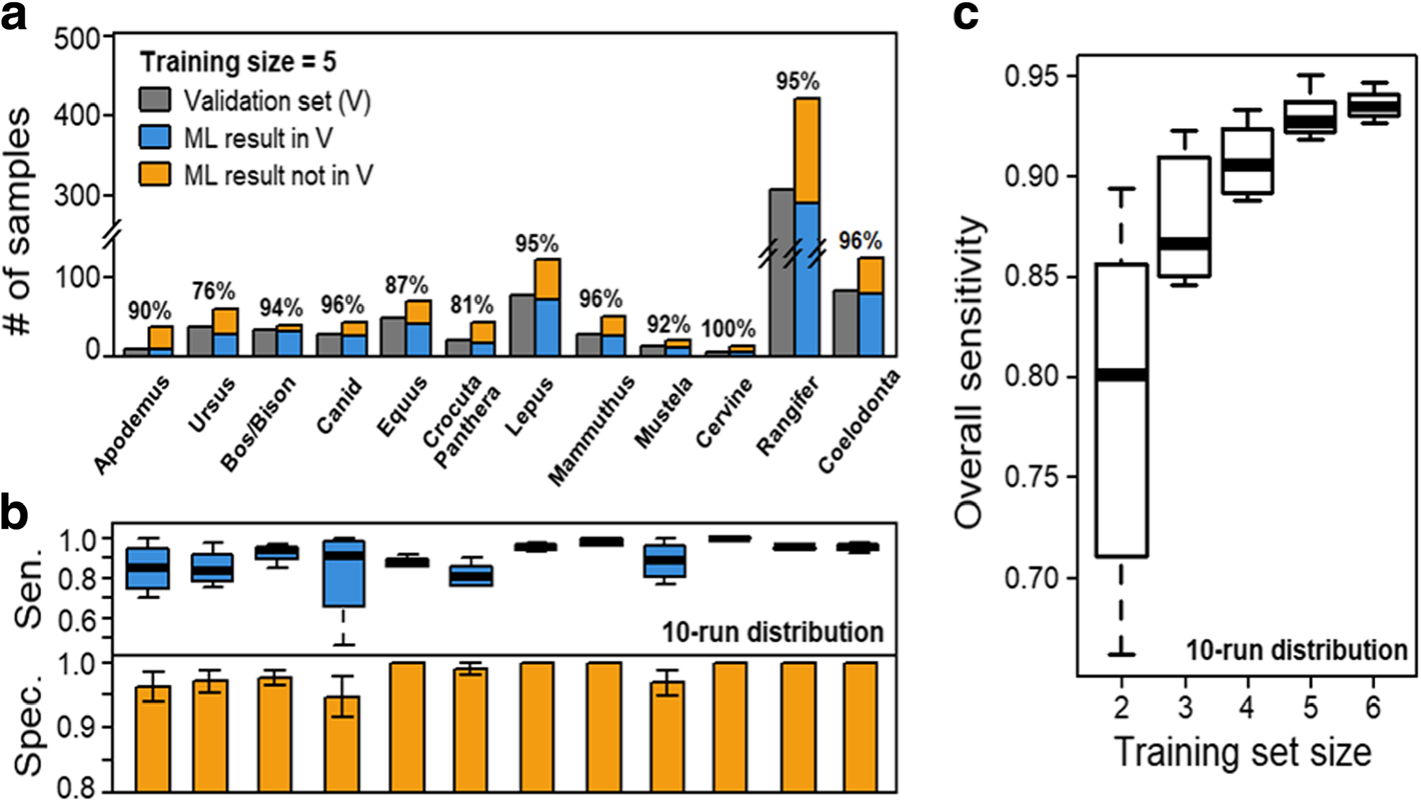
Sensitivity and specificity of semi-supervised learning showing (**a**) a comparison of samples identified by ML to the validation set (percentages of correctly identified samples (i.e. sensitivity) indicated above the bars for each species), (**b**) sensitivity (sen.) and specificity (spec.) of semi-supervised learning (training set = 5) over 10 ML runs shown in box-and-whisker plots (specificity scores were presented as bars due to zero standard deviations of most taxa; bars and error bars represent the mean and standard deviation) and (**c**) the effect of training set size on the sensitivity across many taxa

Any classification method faces the trade-off between sensitivity (i.e. not missing true positives) and specificity (i.e. not including false positives). To assess the sensitivity of our classifier, we compared the output with a validation set published by Buckley et al. (2017) using manually selected biomarkers. For most taxa, ML were able to discover > 90% samples of the validation set (Fig. 4a). Notably, sensitivity reached ~ 95% for *Bos/Bison*, *Lepus*, *Cervine*, *Rangifer*, *Mammuthus* and *Coelodonta*. We repeated the algorithm ten times with randomised starting sets of size = 5 and observed consistent performances (Fig. 4b, blue boxes). ML also identified previously unannotated samples (Fig. 4a yellow bars). To test for false positives within these samples, we manually checked the outputs of ten ML runs and confirmed that the error was within 5% for *Apodemus, Ursus, Bos/Bison, Canid, Crocuta/Panthera* and *Mustela* and is zero for other taxa (Fig. 4b).

Current runs of ML were based on training sets of five samples per taxon. We next investigated the effect of training-set sizes on the accuracy of the ML output and then repeated the ML with a training set of *n* = 2, 3, 4, 5 or 6 samples per taxon. For each size of *n*, 10 runs of supervised ML (each consisting of 8 cycles) were performed. We observed that as the size of training set increases, higher sensitivity was achieved at the end of the 8-cycle runs. Notably, the gain in performance diminished after *n* = 5, which indicates that five samples per species is a reasonable choice for a training set.

### Machine learning outperforms PCA and hierarchical clustering

**Fig. 5:**
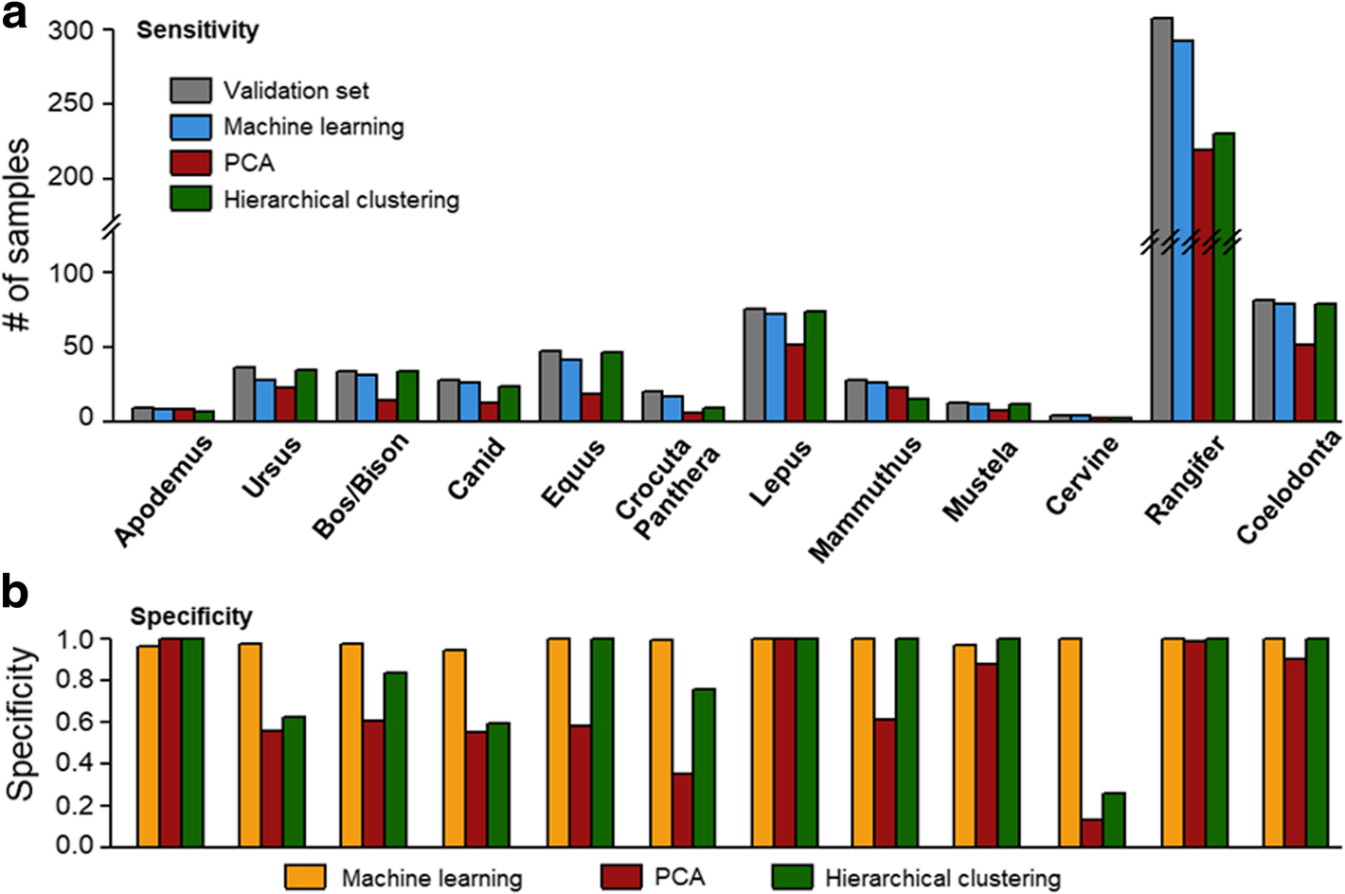
Comparison between ML, PCA and hierarchical clustering showing (**a**) a comparison of sensitivity, indicated by numbers of successfully classified samples within the validation set and (**b**) a comparison of specificity, indicated by proportions of positives discovered by ML but outside the validation set

Given a suitable training set, machine learning (ML) was able to identify species at high discovery rates with few false positives. We next compared its performance with alternative methods such as multivariate analysis and clustering. Principal component analysis (PCA) is a widely used multivariate analysis where the original data is transformed into orthogonal principal components with reduced dimensions. To classify samples, we first calculated the centres of weight for each taxon in the validation set using the first five principal components. Samples were then classified into the nearest centre, given that the distance is within a certain threshold. We screened a range of thresholds to find the optimal value that gives the smallest error (i.e. sum of false positives and negatives). While PCA was able to achieve good sensitivity for *Apodemus* and *Mammuthus* (Fig. 5a), and good specificity for *Apodemus, Lepus, Rangifer* and *Coelodonta* (Fig. 5b), its performance for other species was much less satisfactory.

To test for hierarchical clustering, we computed the distance matrix based on Euclidean distances between binary vectors and constructed the hierarchical tree. The tree was cut down into *n* clusters for a given parameter *n*. We screened the parameter *n* from 10 to 200 and observed optimal performance at *n* = 69. For most taxa, hierarchical clustering achieved similar sensitivity to ML and slightly higher sensitivity for *Equus* (Fig. 5a). However, its relatively lower specificity in *Ursus, Bos/Bison* and *Canid* indicates that it might be prone to false discoveries (Fig. 5b). In addition, the results for PCA and hierarchical clustering represent the best-case scenario since we screened for the optimal parameters against the validation set. In reality, it is rarely achievable since the validation set would be unknown to the user. Therefore a considerable amount of manual work would be required for parameter optimisation. In contrast, machine learning was able to run on a small training set and achieve similar or higher performances.

### Systematic identification of biomarkers

**Fig. 6:**
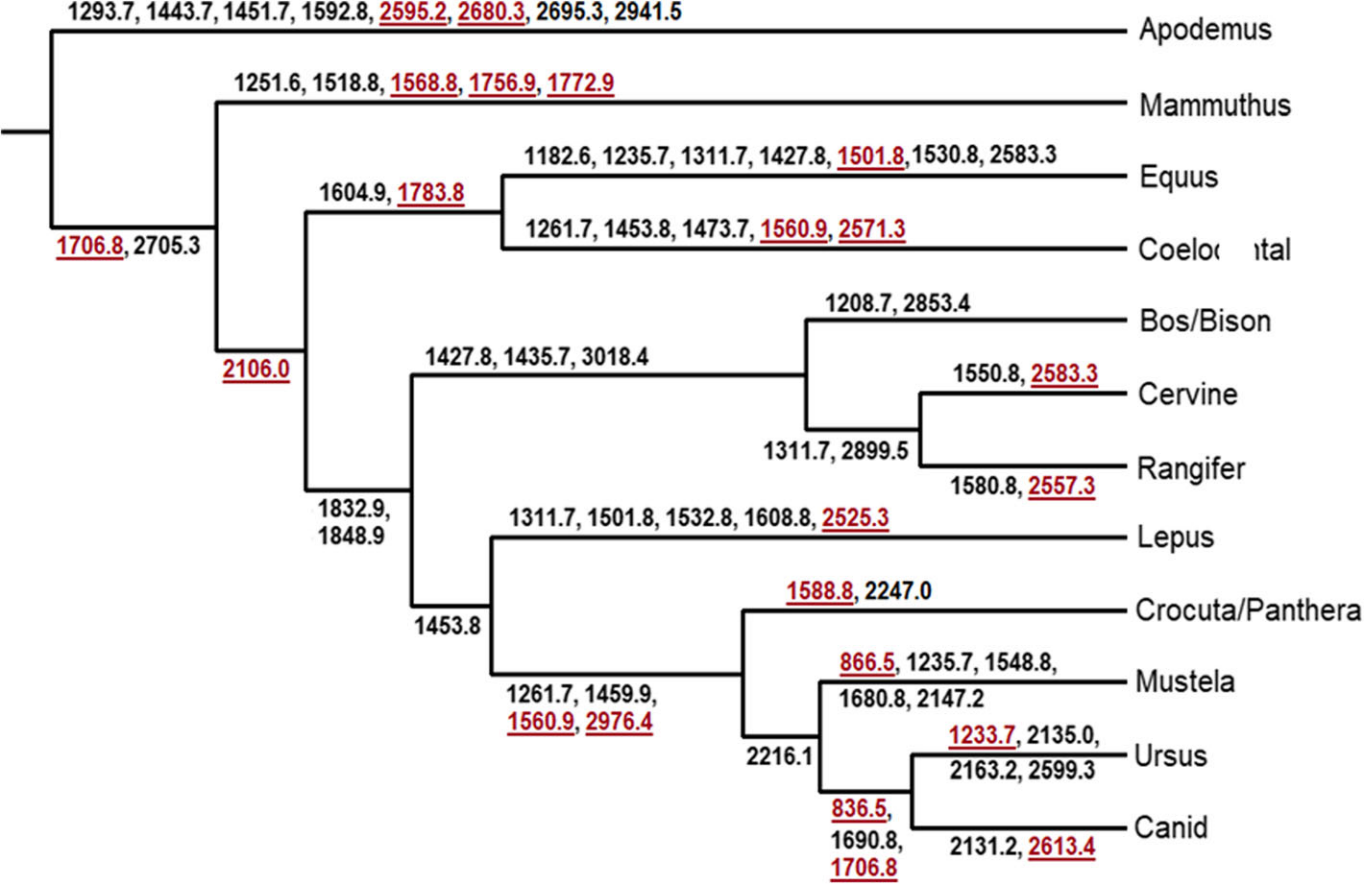
Phylogeny and biomarker discovery based on ML results, created by hierarchical clustering on centres of taxa; biomarkers that separate branches at > 90% accuracy were marked (novel biomarkers highlighted in red)

The drawback of machine learning is that its logic is difficult to interpret since the final decision on species assignment is voted by numerous decision trees. To obtain a simplified view of the classification results, we also investigated which biomarkers can be used to separate species or higher taxonomic groups. We first constructed the phylogenetic tree based on centres of each taxon in the ML output, which produced a topology largely consistent with that expected for the megafauna (e.g., individual groupings of Carnivora, Artiodactyla and Perissocatyla. However, some of the deeper associations were clearly inconsistent with known relationships, such as the lagomorph (*Lepus*) being with the carnivores, and the deep rooting of the rodent *Apodemus*). At each tree node, we searched for biomarkers that can separate the two branches with accuracy > 90%. In addition to previously known biomarkers, we identified a number of new biomarkers that can be used to separate taxonomic groups (Fig. 6).

## Discussion

In this study, we used machine learning (ML) to establish the pipeline for automated species identification from PMF data. The main issue of using simple probabilistic classifiers is the potentially limited performance due to small available training sets. Therefore, we developed an ensemble algorithm based on iterations of random forest that progressively expands the training set and learns towards the final classification scheme. In each cycle, we chose decision trees over support vector machines (SVM) or neural networks for their fast training speed and easy interpretation, given that using SVM as tree constructor yields similar classification results to decision trees (Additional file 2: Figure S3). We initially included closely related species such as *Crocuta* and *Panthera* or *Alopex*, *Vulpes* and *Canis*. However, ML failed to accurately classify some of these species (Additional file 2: Figure S2). Pooling closely related species significantly improved ML performance. Using pooled species as input, we were able to identify > 85% of the samples at family/subfamily level with low false discovery rates. Parameters used in the algorithm were arbitrary rather than optimised since optimisation increases the chance of overfitting. Nevertheless, a scan over various combinations of parameters confirmed the robustness of this approach as long as arbitrary parameters are not of extreme values (Additional file 2: Figure S4). ML differs from clustering methods in the way that it is intrinsically selective towards certain markers since majority voting almost always favours some markers over others, whereas clustering methods usually treat markers with equal weights. Higher performance of ML indicates that using differential weights on markers could be important for distinguishing low level taxonomic groups, which agrees with previous work on keratin for species identification [3].

This approach does not have the support of sequence information, which allows for the confirmation of homology between different markers. One issue is that of PTMs shifting the *m/z* of the peptides being studied. In the case of deamidation, affecting peptides that contain asparagine and glutamine residues, this is relatively predictable and managed by including allowance for the + 1 shift per affected residue (rarely more than 2 or 3 per peptide). In the case of oxidation, for the most part this is a frequent occurrence on collagen’s many proline (and lysine) residues but it is a biological phenomenon not strictly related to decay. However, the oxidation of methionine residues is known to occur by laboratory decay in proteins, but this is a rare amino acid in collagen (e.g., [28]), with the only known exception of one of the manually proposed markers being in one species of marine mammal [29]) and therefore not considered problematic in this study.

The main advantage of this machine learning approach is that it allows for the relaxation of the manual screening criteria that were previously employed to reduce time wasted on manual study of poorer spectra. It is also particularly convincing that there is a very low false positive score for a study of this nature. However, by including an indication of how likely a sample belongs to a taxon (e.g., the similarity score proposed in Fig. 2c; Additional file 5: Table S4), it would allow the user to manually check the most likely spectra to have been falsely identified.

## Conclusion

Here we developed a machine learning approach for automated species identification that vastly reduces the manual work required for analysing high-throughput collagen PMF data of ancient bone samples. This method was able to reach taxonomic resolution at family/sub-family levels within the vertebrata which would provide useful information for ancient samples where DNA was unavailable.

## Additional files

Additional file 1: **Table S1.** Reference peaks for calibration. (DOCX 340 kb)

Additional file 2: **Supplementary figures - Figure S1).** Annotated partial spectra showing approach to distinguishing adjacent peaks from isotopic effects, **Figure S2**). Plots showing the sensitivity and specificity of semi-supervised learning including *Vulpes, Alopex, Canis, Crocuta* and *Panthera with comparison to validation set*, **Figure S3**). Plots of the number of identifications by different algorithms used to construct trees, and **Figure S4**). Plots of the results from variation in parameter scan. (DOCX 340 kb)

Additional file 3: **Table S2.** Binary data matrix of 6,805 PMF spectra. (XLSX 16309 kb)

Additional file 4: **Table S3.** Outputs from Machine Learning cycles. (XLSX 1255 kb)

Additional file 5: **Table S4.** Similarity scores assigned to each identification. (XLSX 364 kb)

## Abbreviations

MALDI-ToF: Matrix Assisted Laser Desorption Ionization
ML: Machine learning
PMF: Peptide mass fingerprint
