# Supplementary material for: Semi-supervised machine learning for automated species identification by collagen peptide mass fingerprinting
Source: BMC Bioinformatics. 2018 Jun 26;19:241. doi: 10.1186/s12859-018-2221-3 (PMC6019507; doi:10.1186/s12859-018-2221-3)
Supplement: Supplementary file 2 — Supplementary figures - Figure S1). Annotated partial spectra showing approach to distinguishing adjacent peaks from isotopic effects, Figure S2). Plots showing the sensitivity and specificity of semi-supervised learning including Vulpes, Alopex, Canis, Crocuta and Panthera with comparison to validation set, Figure S3). Plots of the number of identifications by different algorithms used to construct trees, and Figure S4). Plots of the results from variation in parameter scan. (DOCX 340 kb) [file 12859_2018_2221_MOESM2_ESM.docx]

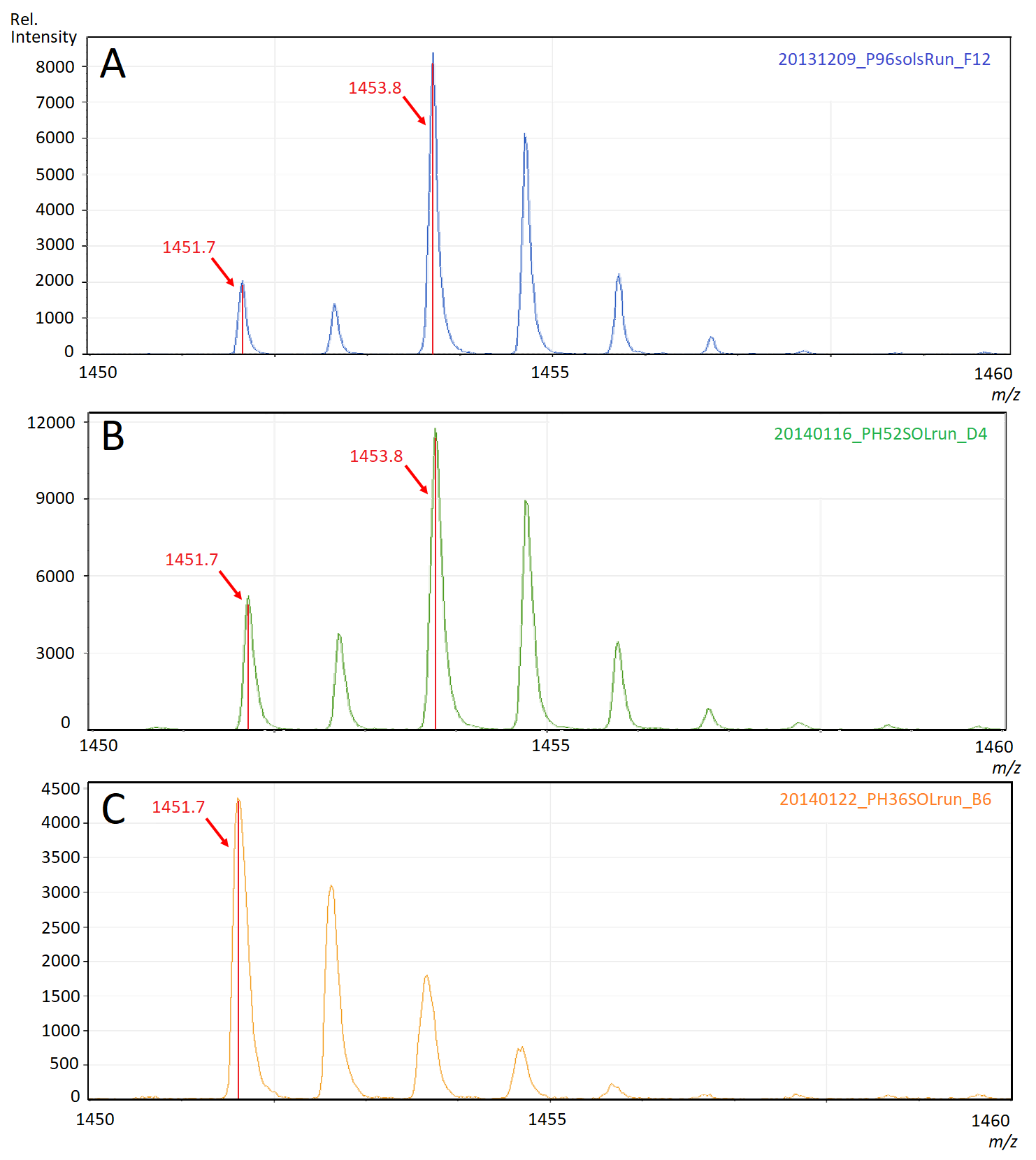


Supplementary Figure 1 – Annotated partial spectra showing approach to distinguishing adjacent peaks from isotopic effects. Peaks that are over 1.5 times lower than its +1 peak and follow the decreasing isotopic trend from its -1 location were labelled as the isotope of an upstream peaks. (A) (B) Examples of isotopic effects of two separate peaks. (C) One single peak.


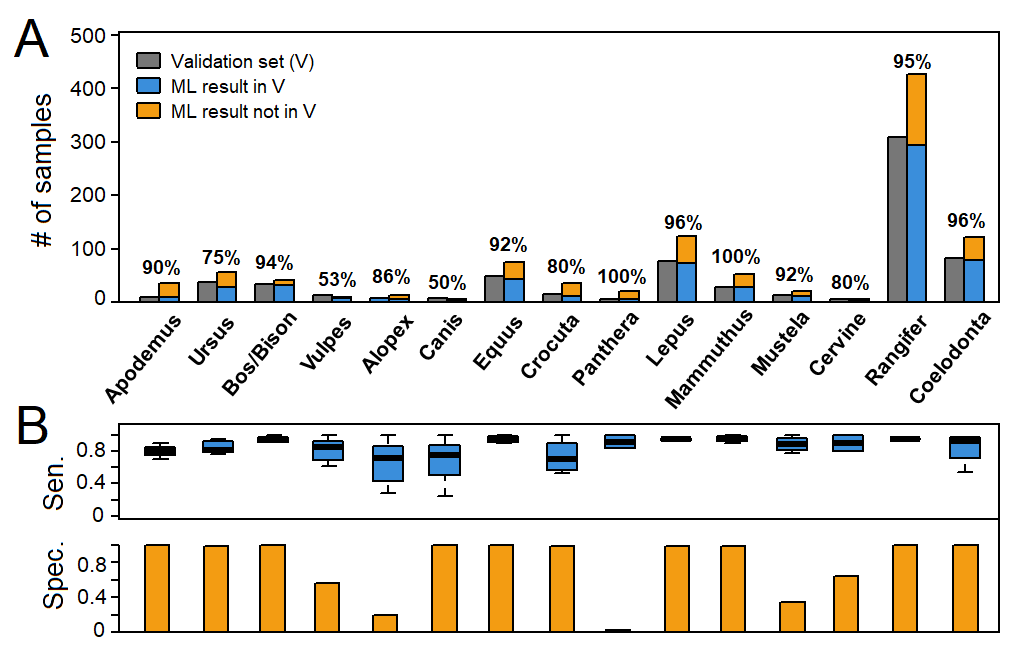


Supplementary Figure 2 – Plots showing the sensitivity and specificity including *Vulpes, Alopex, Canis, Crocuta* and *Panthera*. (A) Comparison of samples identified by machine learning (ML) to the validation set. Percentages of correctly identified samples (i.e. sensitivity) were indicated above the bars for each species. (B) Sensitivity (sen.) and specificity (spec.) of supervised learning (training set = 5). Distribution of sensitivity scores over 10 ML runs were shown in the box-and-whisker plot. Specificity scores were presented as bars due to zero standard deviations of most taxa. Bars and error bars represent the mean and standard deviation of specificity over 10 ML runs.


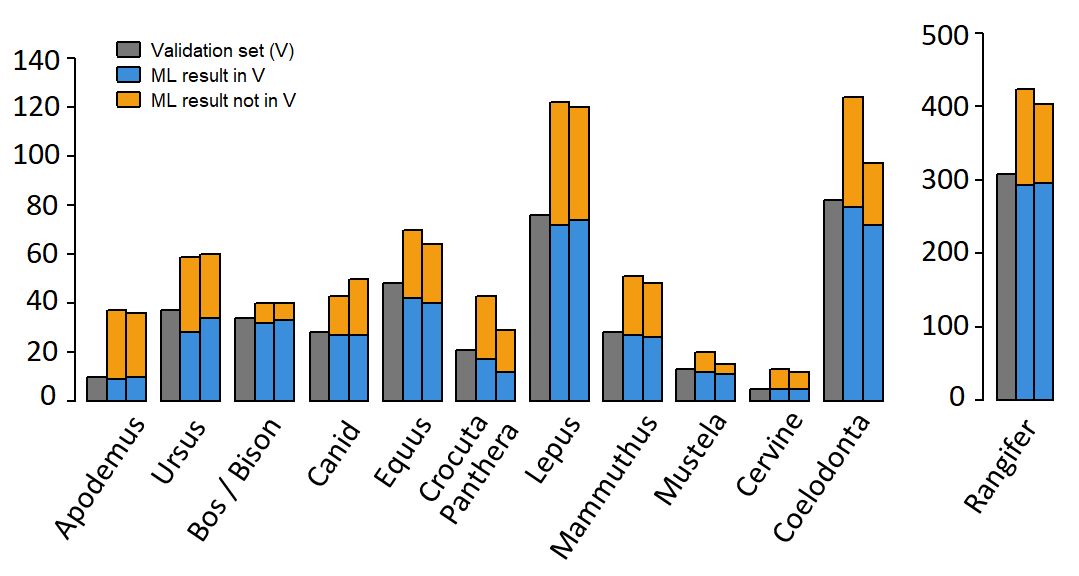


**Supplementary Figure 3 – Plots of the number of identifications by different algorithms used to construct trees (ID3 vs SVM) makes little difference on overall classification output.** Three bars in each set of bars represent number of samples in validation set, identified by ML using ID3 and identified by ML using SVM. SVM were performed by the e1071 package [1] in R without using parameter screening. Other parameters were identical to the original algorithm. Results suggest little difference between ID3 and SVM.


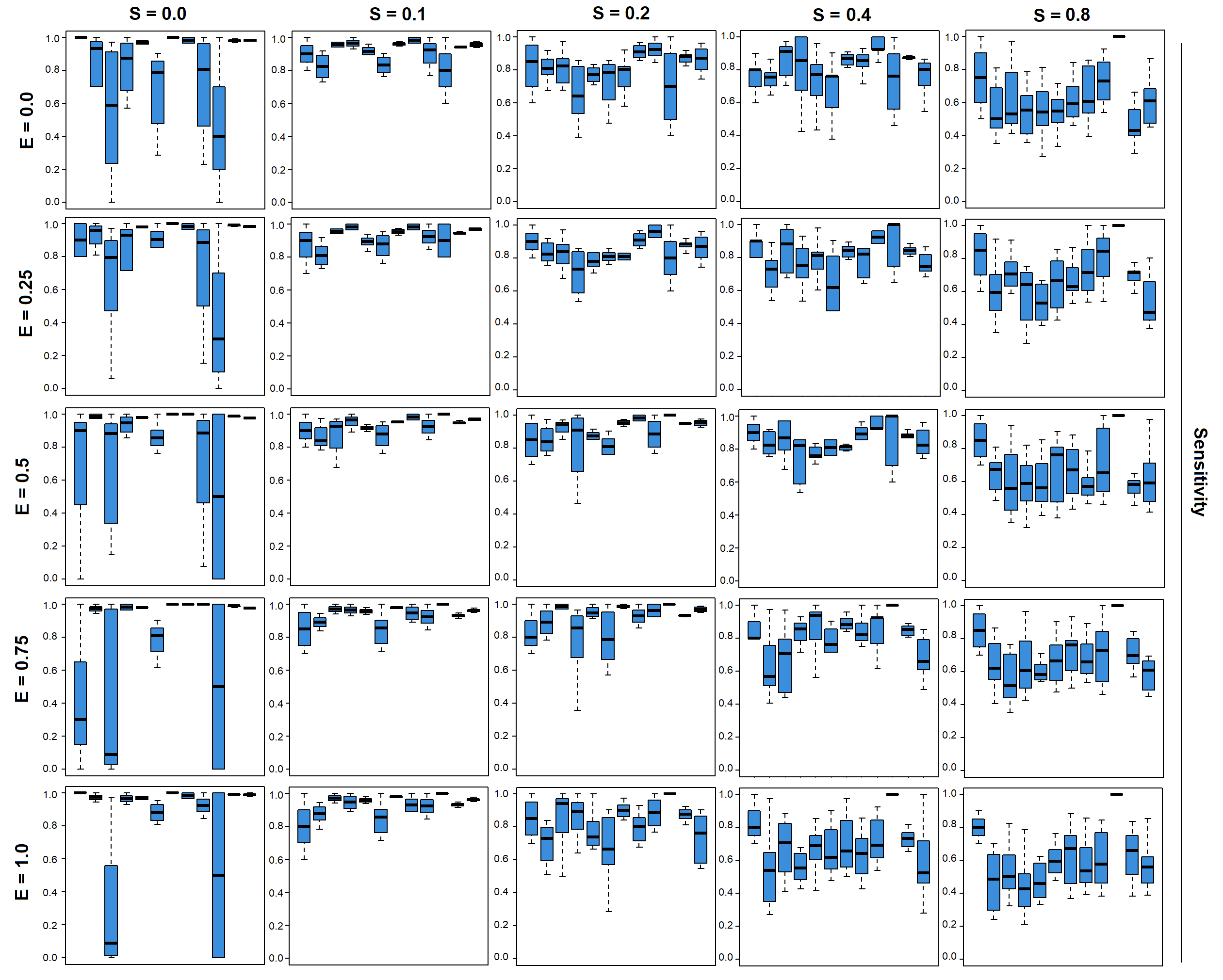


**Supplementary Figure 4 – Plots of the results from variations in parameter scan.** Two parameters were scanned: *E* – penalty for non-unique peaks (used to compute characteristic vectors in Section 2.2; default =0.5) and *S* – cut-off of similarity score below which dissimilar samples were dropped (default = 0.2). *E* has significantly less impact on the ML accuracy than *S*. Extreme values such as *E*=0.0 (i.e. do not penalise peaks that are present in other taxa), *E*=1.0 (i.e. only rely on peaks unique to a given taxon), *S*=0.0 (i.e. do not remove any dissimilar spectra) or *S*=0.8 (i.e. remove top 80% of the most different spectra) tend to result in bad classification accuracy.

**References**

[1] David Meyer , Technische Universität Wien. (2001) Support Vector Machines. The Interface to libsvm in package e1071. Online-Documentation of the package e1071 for R. CRAN package.
